# Phonon Transport and Thermoelectric Properties of Imidazole-Graphyne

**DOI:** 10.3390/ma14195604

**Published:** 2021-09-27

**Authors:** Yanyan Chen, Jie Sun, Wei Kang, Qian Wang

**Affiliations:** 1 School of Materials Science and Engineering, Peking University, Beijing 100871, China; yanyanchen@pku.edu.cn (Y.C.); sunjie6@pku.edu.cn (J.S.); 2 Center for Applied Physics and Technology, HEPDS, College of Engineering, Peking University, Beijing 100871, China; weikang@pku.edu.cn

**Keywords:** pentagon-based 2D material, thermal conductivity, thermoelectric properties, anharmonicity

## Abstract

The pentagon has been proven to be an important structural unit for carbon materials, leading to different physical and chemical properties from those of hexagon-based allotropes. Following the development from graphene to penta-graphene, a breakthrough has very recently been made for graphyne—for example, imidazole-graphyne (ID-GY) was formed by assembling experimentally synthesized pentagonal imidazole molecules and acetylenic linkers. In this work, we study the thermal properties and thermoelectric performance of ID-GY by combining first principle calculations with the Boltzmann transport theory. The calculated lattice thermal conductivity of ID-GY is 10.76 W/mK at 300 K, which is only one tenth of that of *γ*-graphyne (106.24 W/mK). A detailed analysis of the harmonic and anharmonic properties, including the phonon group velocity, phonon lifetime, atomic displacement parameter, and bond energy curves, reveals that the low lattice thermal conductivity can be attributed to the low Young’s modulus, low Debye temperature, and high Grüneisen parameter. Furthermore, at room temperature, ID-GY can reach a high *ZT* value of 0.46 with a 5.8 × 10^12^ cm^−2^ hole concentration, which is much higher than the value for many other carbon-based materials. This work demonstrates that changing structural units from hexagonal to pentagonal can significantly reduce the lattice thermal conductivity and enhance the thermoelectric performance of carbon-based materials.

## 1. Introduction

Thermoelectric materials that can convert waste heat to electricity based on the Seebeck effect have aroused great attention in the energy field. The conversion efficiency of thermoelectric materials is evaluated by a dimensionless figure of merit (*ZT*), *ZT* = *S*^2^*σT*/(*k_e_* + *k_l_*), which depends on the synergetic effect of the Seebeck coefficient (*S*), electrical conductivity (*σ*), absolute temperature (*T*), electronic thermal conductivity (*k_e_*), and lattice thermal conductivity (*k_l_*). However, most commercial thermoelectric materials are based on elements that are relatively scarce and/or toxic, such as Bi_2_Te_3_ [1], PbTe [2], and Sb_2_Te_3_ [3]. Therefore, there is a need to find other earth-abundant and environmentally friendly materials with a good thermoelectric performance.

For this, carbon-based materials can be candidates because of their nontoxicity, light weight, low cost, and high compatibility. More importantly, the lattice thermal conductivity of carbon materials can vary within a huge range of five orders of magnitude depending on the atomic configuration [4]. Usually, materials with a low lattice thermal conductivity are desirable in thermoelectric applications for energy conversion. It has been found that carbon materials can reach very low lattice thermal conductivities and exhibit a good thermoelectric performance [5,6,7]. For instance, Yan’s group reported that the thermoelectric properties of carbon nanotubes (CNTs) can be significantly enhanced by changing their morphology to CNT bulky papers with Ar plasma treatment. The *ZT* value of CNT bulky papers is increased from 0.01 for pristine CNTs to 0.4 for Ar plasma-treated CNTs [8]. Chen et al. found that the thermal conductivity of single-walled carbon nanotube (SWNT)/polyaniline (PANI) hybrid film is only 0.43 W/mK and that the *ZT* value reaches 0.12 at room temperature, remarkably higher than that of either of the individual components of the composite [9]. Meanwhile, for two-dimensional (2D) carbon materials, graphene is a typical representative and exhibits a high electrical conductivity, which is one of the essential requirements for thermoelectric materials. However, graphene possesses an ultra-high lattice thermal conductivity (3151.53 W/mK at 300 K) [10] with a low Seebeck coefficient (about 100 μV/K at 300 K) [11] because of its gapless band structure and strong *sp^2^* covalent bonds, hindering its application in the thermoelectric field. As an allotrope of graphene, the recently synthesized *γ*-graphyne [12] has provided a new possibility for the application of carbon-based materials in the thermoelectric field due to its high Seebeck coefficient of 690 μV/K [13] and low *k_l_* of 106.24 W/mK [14], which are superior to the corresponding values of graphene.

On the other hand, it has been found that changing structural units can not only change the geometrical structures of materials but also significantly change the values of the lattice thermal conductivity. For instance, the lattice thermal conductivity of penta-graphene is found to be 645 W/mK at room temperature [15], much lower than the 3151.53 W/mK of graphene [10]. When going from 2D carbon sheets to one-dimensional (1D) and three-dimensional (3D) carbon structures, a similar trend also exists. For 1D carbon, the lattice thermal conductivity of a pentagon-based nanotube is only 95.87 W/mK, which is less than one tenth of the value of (6,6) a carbon nanotube with a similar tube radius [16]. For penta-diamond, which is a new 3D carbon allotrope consisting of five-membered rings, the lattice thermal conductivity is 490.88 W/mK at room temperature [17], much lower than that of diamond (2664.93 W/mK). These results clearly show that the pentagonal unit can effectively modulate the thermal transport of carbon materials. Very recently, we proposed a 2D pentagon-based derivative of graphyne, imidazole-graphyne [18], named ID-GY, which has a direct band gap of 1.10 eV, a low Young’s modulus, and strong refraction near infrared (IR), with potential applications in nanoelectronics and optical devices. ID-GY could be formed by assembling experimentally synthesized five-membered imidazole molecules with acetylenic linkers, as exhibited in our previous work [18]. In this work, we further study the thermal transport and thermoelectric properties of ID-GY.

## 2. Computational Methods

Geometry optimization and electronic band structure calculation are carried out using density functional theory (DFT), as implemented in the Vienna ab initio simulation package (VASP) [19], using the projector augmented wave (PAW) method [20,21]. The Perdew–Burke–Ernzerhof (PBE) functional [22] within the generalized gradient approximation (GGA) [23] is used to treat the exchange–correlation interaction of electrons, while the Heyd–Scuseria–Ernzerhof hybrid functional (HSE06) [24] is used for more accurate band-structure calculations. The kinetic energy cutoff of wave function is set to 520 eV, and the Monkhorst–Pack [25] *k*-point, with a grid density of 2π × 0.02 Å^−1^, is used to sample the Brillouin zone for integration in the reciprocal space. All atomic positions are fully optimized with convergence thresholds of 10^−8^ eV and 10^−6^ eV/Å for the total energy and force component, respectively. During the calculations of geometry optimization and band structure, 2D periodic boundary conditions along the *x* and *y* directions are applied to ID-GY, while a vacuum region of 16.59 Å is set along the *z* direction to exclude the mirror interactions between adjacent images.

The electrical transport properties, including the Seebeck coefficient, electrical conductivity, and electronic thermal conductivity are calculated using BoltzTraP2 software [26]. The lattice thermal conductivity is calculated using the ShengBTE package [27]. The second and third interatomic force constants (IFCs) are obtained based on a 2 × 2 supercell using phonopy software [28] and thirdorder.py code [27], respectively. To calculate the anharmonic IFCs, we include the interactions up to the ninth-nearest neighbor atoms with the cutoff radius of 4.72 Å. The *q*-grids of 40 × 40 and the thickness of 3.4 Å are chosen to solve the phonon Boltzmann transport equation.

## 3. Results and Discussion

### 3.1. Phonon Spectrum and Band Structure

**Figure 1:**
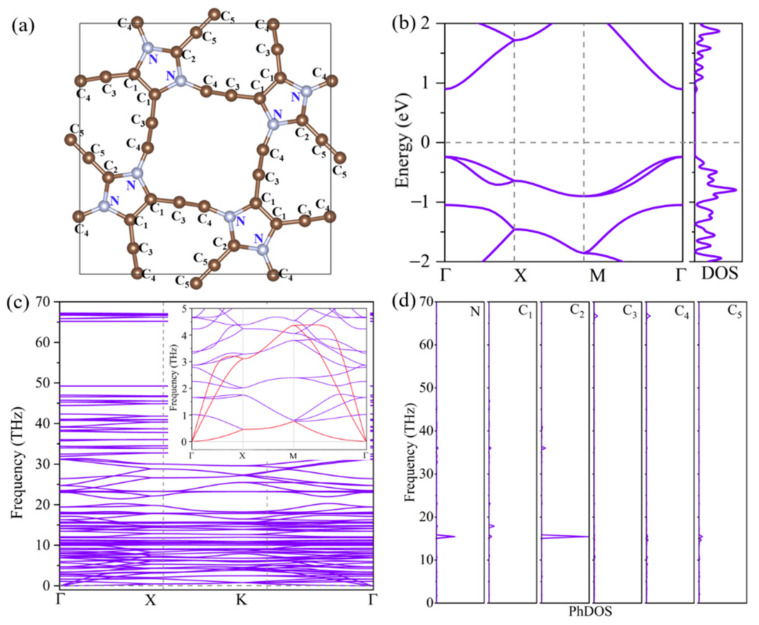
(**a**) Geometric structure, (**b**) electronic band structure, (**c**) phonon spectrum, and (**d**) partial phonon density of states (PhDOS) of ID-GY.

ID-GY crystals in a tetragonal unit cell with the lattice constants of *a* = *b* = 12.14 Å containing 32 carbon and 8 nitrogen atoms in the unit cell with the space group symmetry of P4/mbm (No. 127). Chemically nonequivalent atoms are marked in Figure 1a, where *C_1_*, *C_2_*_,_ and *N* atoms are in *sp*^2^ hybridization while *C_3_*, *C_4_,* and *C_5_* atoms are in *sp* hybridization. Unlike *γ*-graphyne, composed of hexagonal units, ID-GY is composed of pentagonal units connected by acetylenic linkers. Compared with the highly symmetric *γ*-graphyne, the complex geometric structure and hybridized bonding in ID-GY make it a promising material with a low lattice thermal conductivity, just like graphyne and graphdiyne [29,30].

The phonon dispersion spectrum of ID-GY along the high symmetry *k*-point path (*Γ*-*X*-*M*-*Γ*) in the first Brillouin zone is shown in Figure 1c. All the vibrational modes are real in the entire Brillouin zone, confirming that ID-GY is dynamically stable. Since both carbon and nitrogen are light atoms, the highest frequency in the phonon spectrum reaches 67 THz. In addition, one can see that there is a large phonon band gap (about 15 THz) in the high-frequency region, and the corresponding phonon density of states (PhDOS) (Figure 1d), indicate that the high frequency can be attributed to the *C_3_* and *C_4_* atoms. The bond length of *C_3_*-*C_4_* is 1.23 Å, showing the characteristics of alkyne bonds. Because the large portion of heat is carried by low-frequency phonons, especially the acoustic phonons, the low-frequency region of the phonon spectrum is magnified, and the acoustic phonon branches are highlighted in red. The longitudinal acoustic (LA) and transverse acoustic (TA) branches of ID-GY are linear when the wave vector *q* is close to the *Γ* point, while the out-of-plane acoustic (ZA) branch exhibits parabolic dispersion, which is a characteristic of monolayer 2D materials [31]. The highest frequency of the acoustic phonon is relatively low (<5 THz), lower than that of *γ*-graphyne (about 8 THz) [14]. The low frequency of the acoustic phonons is associated with a low acoustic Debye temperature, as discussed in the following paragraph. Moreover, there is a strong overlap between the acoustic and low-frequency optical branches. These characteristics indicate that the lattice thermal conductivity of ID-GY might be low.

To examine the mechanical stability of ID-GY, the elastic constants were calculated and are listed in Table 1. It is obvious that ID-GY satisfies the Born–Huang criteria [18,32] for 2D tetragonal materials—namely, *C_11_* > 0, *C_66_* > 0 and *C_11_* > *C_12_*. The Young’s modulus *Y*, Poisson’s ratio *ν*, bulk modulus *B*, and shear modulus *G* were also calculated and are presented in Table 1. It was found that the stiffness (122.20 N/m) of ID-GY is only half that of graphene (342 N/m) [33], owing to weak in-plane bonds. Moreover, the sound velocity, which is usually used to measure the speed of phonons propagating through the lattice, can be determined from bulk modulus *B* and shear modulus *G* by the following formulas [34]: longitudinal sound velocity *v_l_* = $\sqrt{\frac{B+G}{\rho }}$, transverse sound velocity *v_t_* = $\sqrt{\frac{G}{\rho }}$, and average sound velocity vs. = 1/$\sqrt[{3}]{\frac{1}{3}(\frac{1}{v_{l}^{2}}+\frac{2}{v_{t}^{2}})}$, where *ρ* is the mass density. Based on the sound velocity, we obtained the Debye temperature using *θ_D_* = $\frac{\hbar v_{s}}{k_{B}}{\left(\frac{4\pi N}{S}\right)}^{1/2}$, where *N* is the number of atoms in the cell and *S* is the area of the unit cell. Debye temperature measures the temperature above which all modes begin to be excited; therefore, a high *θ_D_* indicates weak three-phonon scattering and hence a high *k_l_*. The calculated Debye temperature of ID-GY is 647 K, which is much lower than the corresponding value of 805 K of *γ*-graphyne. Consequently, it is natural to expect that ID-GY possesses a lower lattice thermal conductivity than *γ*-graphyne.

To study the electrical transport property, we calculated the band structure of ID-GY. As shown in Figure 1b, ID-GY exhibits semiconducting electronic features with a direct bandgap value of 1.10 eV. Compared with the bandgap (0.47 eV) of *γ*-graphyne [14], the larger bandgap can effectively overcome the high-temperature bipolar conduction problem, benefitting thermoelectric performance [35]. Moreover, the sharp conduction band and valence band around the *Γ* point suggest low carrier effective masses and a possible large carrier mobility. It is worth noting that the valence band maximum (VBM) is doubly degenerated, leading to a sharp density of states (DOS). The high degeneracy of the valence band and the sharp DOS would enhance the Seebeck coefficient of p-type ID-GY, as is the case with bilayer MoS_2_ [36].

### 3.2. Thermal Transport Properties

**Figure 2:**
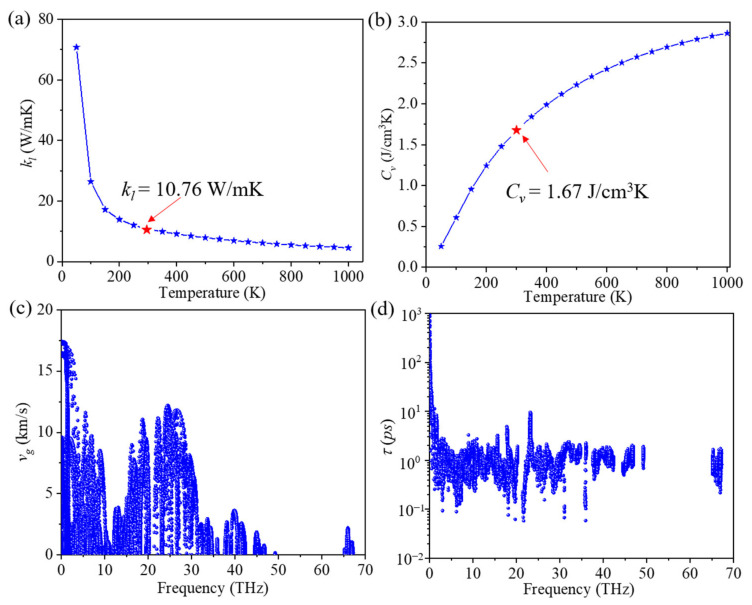
(**a**) Lattice thermal conductivity (*k_l_*), (**b**) phonon volumetric-specific heat (*C_v_*), (**c**) group velocity (*v_g_*), and (**d**) phonon lifetime (*τ*) of ID-GY.

The lattice thermal conductivity (*k_l_*) of ID-GY was calculated for different temperatures. As shown in Figure 2a, the lattice thermal conductivity of ID-GY is 10.76 W/mK at 300 K, which is two orders of magnitude lower than that of graphene (3151.53 W/mK) [10] and much lower than that of many other 2D carbon hexagonal structures, including *α*-graphyne (21.11 W/mK) [14], *β*-graphyne (22.3 W/mK) [14], *γ*-graphyne (106.24 W/mK) [14], and graphdiyne (22.3 W/mK) [29], at the same temperature. This shows the importance of structural units in affecting the thermal conductivity of a material. We fitted the relationship of *k_l_* with temperature and found that *k_l_* is proportional to 1/T^1.05^, indicating that the three-phonon scattering is dominant in ID-GY, as is the case with graphene [38] and penta-graphene [15]. This was further confirmed by comparing the scattering rates of three-phonon scattering with those of the isotopic scattering process. The calculated results for these two scattering processes are plotted in Figure S1 in the Supplementary Materials. We found that the three-phonon scattering rates are nearly 100 times larger than the isotopic scattering ones. The calculated cumulative *k_l_* as a function of frequency is plotted in Figure S2, which shows that the phonons with frequencies lower than 20 THz contribute about 85% to the lattice thermal conductivity. Therefore, we focus on the low-frequency phonon branches (<20 THz) in the following discussion.

It is important to understand the reasons for the low *k_l_* of ID-GY. *k_l_* can be expressed in the following form through the summation of the contribution of all of the phonon modes *λ*(*q*, *j*) with the wave vector *q* and branch index *j*:

(1) $$k_{l}=\frac{1}{N}\sum_{\lambda }C_{\lambda }\nu_{\alpha ,\lambda }\nu_{\beta ,\lambda }\tau_{\lambda },$$

where *α* and *β* denote the three directions (*x*, *y,* or *z*) and *λ* is the phonon mode consisting of both wave vector *q* and branch index *j*. $C_{\lambda }$, $\nu_{\alpha ,\lambda }$, and $\tau_{\lambda }$ represent the phonon volumetric-specific heat, group velocity, and phonon lifetime, respectively.

Our calculated phonon specific heat value of ID-GY is 1.67 J/cm^3^K at 300 K. The variation in the phonon volumetric-specific heat with temperature is plotted in Figure 2b. It is worth mentioning that the phonon-specific heat value usually is not different from one material to another [39]. For instance, the phonon-specific heat value of *γ*-GY is 1.68 J/cm^3^K [29], which is almost same as that of ID-GY. The change in group velocity of ID-GY with frequency is given in Figure 2c, which shows that at the long-wavelength limit, the group velocity reaches the highest value of 17.5 km/s, close to that of *γ*-GY (~17.9 km/s) [14]. In the low-frequency region (below 20 THz), the overall group velocity is only slightly lower than that of *γ*-GY. The variation in phonon lifetime with frequency at room temperature is plotted in Figure 2d. The lifetime for most low-frequency phonon modes (0~20 THz) is about 2 *ps*, while that for *γ*-GY is larger than 10 *ps* [14]. Therefore, the short phonon lifetime in ID-GY is the main reason for the low lattice thermal conductivity.

**Figure 3:**
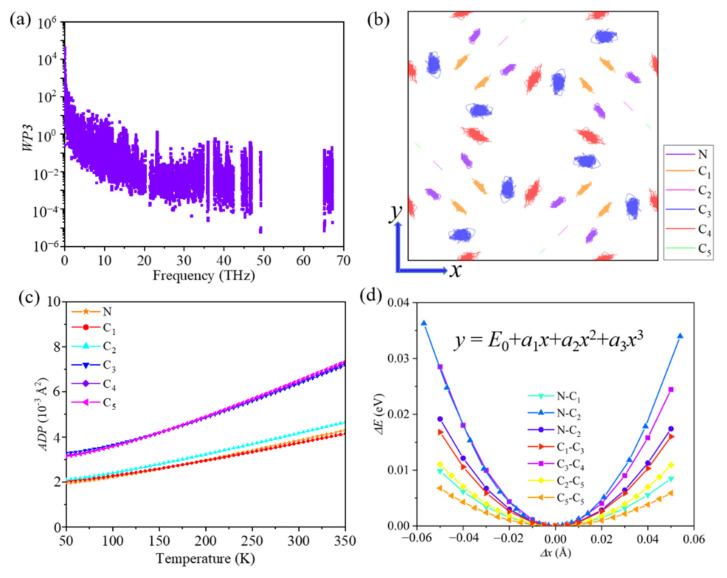
(**a**) Weighted phase space (*WP3*), (**b**) trajectory of the atoms in the *xy*-plane from ab initio molecular dynamics simulations at 800 K, (**c**) atomic displacement parameter (ADP), and (**d**) bond energy curves of ID-GY.

A short phonon lifetime is usually associated with strong phonon scattering. Therefore, we studied the phonon scatterings by carrying out additional calculations based on the scattering mechanism of phonon modes. Three-phonon scattering is the dominant process in the heat transport process and usually depends on two factors: the number of existing scattering channels and the strength of each scattering channel, which can be described by weighted phase space (*WP3*) and mode Grüneisen parameter (*γ*). The weighted phase space of each phonon mode with respect to phonon frequency is plotted in Figure 3a. We found that the value of the *WP3* is relatively large, which results from the large number of atoms per unit cell and the complex geometric structure of ID-GY. Moreover, the Grüneisen parameter, which quantifies the intensity of anharmonic interactions between the phonon branches, is another key factor in determining the phonon lifetime of a system. The average Grüneisen parameter of ID-GY was calculated to be 1.51, which is comparable to that of traditional thermoelectric material PbTe (1.65) [40], suggesting the existence of a strong anharmonicity in ID-GY. 

To find the origin of the strong anharmonicity from the point of view of geometric structure, we plotted the simulated trajectory of atoms in the *xy*-plane at 800 K during an ab initio molecular dynamics (AIMD) simulation. As shown in Figure 3b, the *C*_3_ and *C*_4_ atoms vibrate strongly around their equilibrium positions. The atomic trajectory shows that -*C*_3_≡*C*_4_- pairs in acetylenic linkers are weakly bonded to the pentagonal rings. These conclusions are further confirmed by the atomic displacement parameter (ADP) and the bond energy curve, which can provide a visualization of the anharmonicity. As shown in Figure 3c, the ADPs of the *C*_3_ and *C*_4_ atoms are much larger. The bond energy curve shows the vibration of a relative energy change Δ*E* (in eV/per atom) when the bond length changes and reveals the phonon anharmonicity. By fitting the bond energy curve (Figure 3d), we obtain the anharmonic parameters (*a*_3_) for different type of bonds in ID-GY. The data in Table 2 show that the strong anharmonicity in ID-GY mainly originates from the -*C*_3_≡*C*_4_- pairs, which are weakly bonded to the pentagonal rings. The single *N*-*C*_4_ and *C*_1_-*C*_3_ bonds are too weak to yield an inefficient thermal transport by lattice vibration. The inhomogeneous bond environment and large lattice vibrational mismatch between the pentagonal rings and the acetylenic linkers hinder the transport of heat. 

### 3.3. Electrical Transport Properties

**Figure 4:**
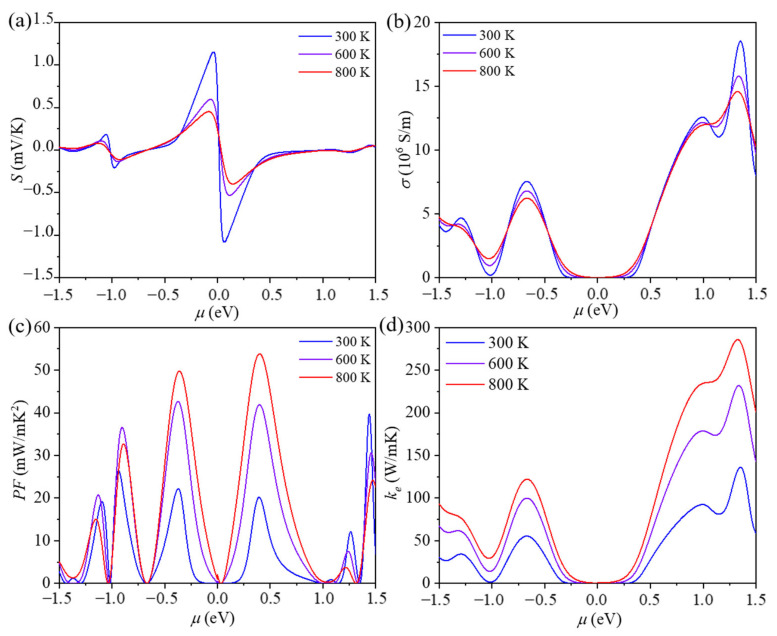
(**a**) Seebeck coefficient (*S*), (**b**) electrical conductivity (*σ*), (**c**) power factor (*PF*), and (**d**) electronic thermal conductivity (*k_e_*) of ID-GY as a function of chemical potential *μ*, respectively.

ID-GY possesses an appropriate band gap and a low lattice thermal conductivity; therefore, it could be a high-performance thermoelectric material. To study the thermoelectric performance of ID-GY, we calculated its electrical transport properties. To this end, the Seebeck coefficient (*S*) was calculated using the BoltzTraP2 software [26]. As shown in Figure 4a, the *S* can reach a peak of 1150 μV/K at 300 K, which is much larger than that of *γ*-graphyne (690 μV/K) [13] owing to the larger bandgap and doubly degenerate valence bands of ID-GY. To obtain the electronic conductivity, the carrier relaxation time (*τ*) is necessary, which can be expressed as:

(2) $$\tau =\frac{\mu \left|m^{*}\right|}{e}=\frac{2\hbar^{3}C}{3k_{B}T\left|m^{*}\right|E_{1}^{2}},$$

where *μ* is the carrier mobility, *C* is the in-plane elastic constant, and *E*_1_ is the deformation-potential constant. The effective mass of carrier m* was calculated from the curvature of the conduction band minimum or valence band maximum by the parabolic fitting of the band edge using the formula m* = $\hbar {\left[\partial^{2}E/\partial k^{2}\right]}^{-1}$. These calculated results are summarized in Table 3.

As expected from the band structure, the effective mass of the carrier would be very low. The calculated value is indeed only 0.11~0.25 *m*_e_, indicating that ID-GY could have a considerably high carrier mobility. The obtained carrier mobilities of ID-GY are 7933 and 1826 cm^2^/Vs for electrons and holes, respectively; accordingly, the electron relaxation time (*τ_e_*) is much longer than the hole relaxation time (*τ_h_*). It is worth noting that although the deformation potential approximation [41] has been widely used for predicting the carrier mobility of new thermoelectric materials [42,43], the carrier mobility is usually overestimated as compared to the experimental result due to the neglect of scattering between the carrier and either the defect or the substrate [44]. Conversely, these scattering processes can also reduce the thermal conductivity. Thus, the overestimated electrical conductivity and overestimated thermal conductivity may cancel each other out to some extent, resulting in a more reliable prediction. 

Based on the carrier relaxation time, the electrical conductivity (*σ*) is obtained and presented in Figure 4b. The electronic conductivity of n-type ID-GY is higher than that of p-type ID-GY due to the longer electron relaxation time. We note that the absolute values of the Seebeck coefficient and electrical conductivity show opposite trends when the chemical potential changes. Thus, to obtain a good power factor (*PF* = *S*^2^*σ*), an optimum chemical potential is needed. The maximum *PF* value for p-type ID-GY is 22.14 mW/mK^2^ at 300 K, while that for p-type ID-GY is 20.18 mW/mK^2^. 

Thermal conductivity is the sum of the lattice thermal conductivity and electronic thermal conductivity. The former is described in Equation (1) and the latter follows the Wiedemann–Frranz law *k_e_* = *LσT*, where the Lorenz number *L* is equal to 2.44 × 10^−8^ WΩ/K^2^ [45]. As shown in Figure 4d, electronic thermal conductivity has a similar tendency to electrical conductivity.

**Figure 5:**
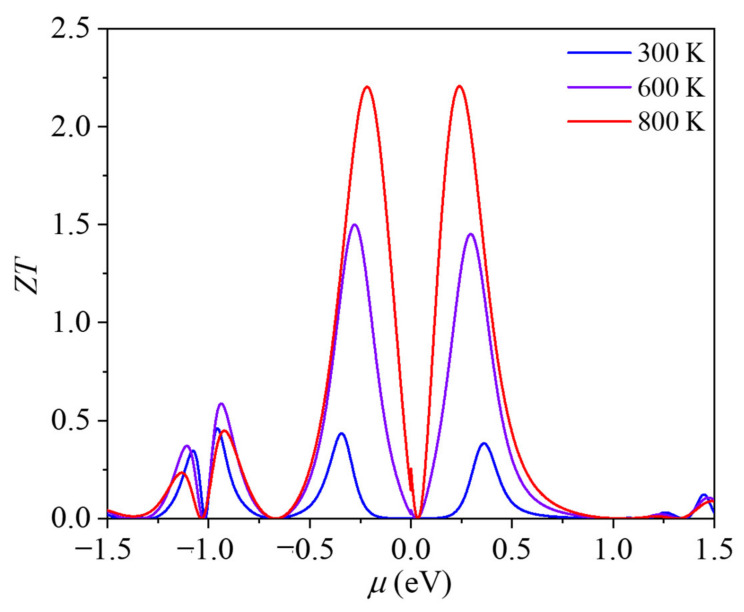
Variation in the thermoelectric figure of merit (*ZT*) of ID-GY with the chemical potential (*μ*).

Finally, the thermoelectric performance of ID-GY was evaluated using the *ZT* value. The variations in our calculated *ZT* values with chemical potential *μ* at different temperatures are plotted in Figure 5. At 300 K, the optimized *ZT* values of ID-GY can reach 0.46 and 0.38 for p-type and n-type with the hole- and electron-doping concentrations of 5.8 × 10^12^ and 1.75 × 10^12^ cm^−2^, respectively, which are higher than the *ZT* values of many other 2D carbon materials [30], including graphene (0.01), *α*-graphyne (0.03), *β*-graphyne (0.12), *γ*-graphyne (0.17), and 6,6,12-graphyne (0.05). While at 800 K, the *ZT* values are 2.20 and 2.21 for p-type and n-type ID-GY. 

## 4. Conclusions

In this work, based on first principle calculations, we investigated the thermal transport and thermoelectric properties of ID-GY, a new pentagon-based 2D material that is constructed by assembling an experimentally synthesized pentagonal imidazole molecule and acetylenic linkers. We showed that the thermoelectric properties of graphyne are significantly improved by changing its structural unit to a pentagonal imidazole molecule. The lattice thermal conductivity was decreased from 106.24 W/mK for *γ*-graphyne to 10.76 W/mK for ID-GY at 300 K, while the Seebeck coefficient was increased to 1150 V/K for ID-GY from 690 μV/K for *γ*-graphyne at room temperature. The mechanism of the low lattice thermal conductivity was further studied by analyzing the group velocity, scattering rate, weighted phase space, and bond energy curve. The mismatched lattice vibration between the pentagonal rings and the linkers in ID-GY resulted in a strong anharmonicity. Moreover, as compared to *γ*-graphyne, the doubly degenerated VBM and larger band gap resulted in a higher Seebeck coefficient in ID-GY. The calculated thermoelectric figure of merit at 300 K was 0.46, suggesting the effectiveness of using the pentagonal structural unit for enhancing the thermoelectric performance of carbon-based materials. 

## Figures and Tables

**Table 1:** Calculated elastic coefficients *C_ij_* (in N/m), Young’s modulus *Y* (in N/m), Poisson’s ratio *ν*, bulk modulus *B* (in N/m), shear modulus *G* (in N/m), longitudinal sound velocity *v_l_* (in km/s), transverse sound velocity *v_t_* (in km/s), average sound velocity vs. (in km/s), and Debye temperature *θ_D_* (in K) for ID-GY. For comparison, the corresponding values for *γ*-graphyne (*γ*-GY) are also listed here.

	*C*_11_	*C*_12_	*C*_66_	*Y*	*ν*	*B*	*G*	*v_l_*	*v_t_*	*v_s_*	*θ_D_*
ID-GY	164.26	83.12	12.79	122.20	0.51	124.69	40.46	17.17	8.5	4.59	647
*γ*-GY	-	-	-	-	0.41 *	122.73 *	77.04 *	18.53	11.51	5.50	805

* Data from ref. [37].

**Table 2:** Fitted anharmonic parameters (*a*_3_) of the bond energy curves for ID-GY.

	*C*_3_ *-C*_4_	*N-C*_4_	*N-C*_1_	*C*_1_ *-C*_3_	*C*_5_ *-C*_5_	*N-C*_2_	*C*_2_ *-C*_5_
*a_3_*	15.41	7.13	5.90	4.28	3.28	0.68	0.60

**Table 3:** In-plane elastic constant *C* (in N/m), deformation-potential constant *E*_1_ (in eV), effective mass of carrier m* (in *m*_e_), carrier mobility *μ* (in cm^2^/Vs), and carrier relaxation time *τ* (in 10^−14^ *s*) of ID-GY at 300 K.

Carrier Type	*C*	*E*_1_	m*	*μ*	*τ*
electron	164.26	4.94	0.11	7932.54	49.56
hole	164.26	4.53	0.25	1826.31	25.93

## Data Availability

The data presented in this study are available on request from the corresponding author.

## Supplementary Materials

The following are available online at https://www.mdpi.com/article/10.3390/ma14195604/s1, Figure S1: the isotopic scattering rates of ID-GY, Figure S2: viaration of the normalized cumulative lattice thermal conductivity with frequency for ID-GY.
