# Supplementary material for: Phonon Transport and Thermoelectric Properties of Imidazole-Graphyne
Source: Materials (Basel). 2021 Sep 27;14(19):5604. doi: 10.3390/ma14195604 (PMC8509738; doi:10.3390/ma14195604)
Supplement: Supplementary file 1 [file materials-14-05604-s001.zip › materials-1389255-supplementary.pdf]

Phonon Transport and Thermoelectric Properties of Imidazole-Graphyne

Yanyan Chen[1,2], Jie Sun[1,2], Wei Kang,[2] and Qian Wang[1,2]*

1 School of Materials Science and Engineering, Peking University, Beijing 100871, China

2 Center for Applied Physics and Technology, HEPDS, College of Engineering, Peking University, Beijing, 100871, China

* Corresponding author: qianwang2@pku.edu.cn

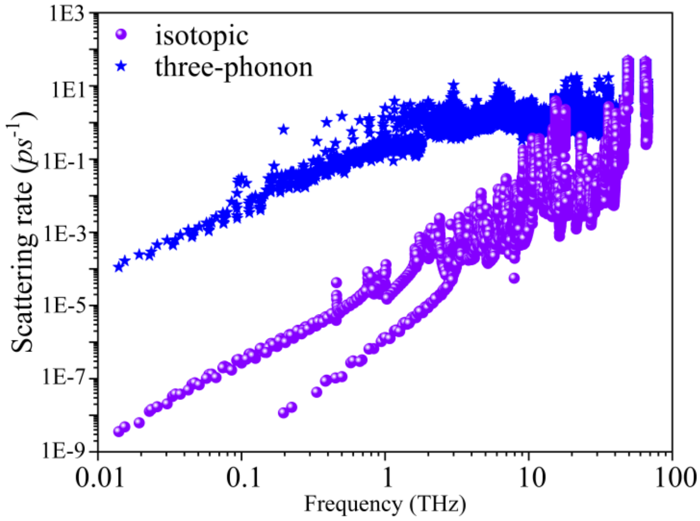

Figure S1. Three-phonon and isotopic scattering rates of ID-GY at room temperature.

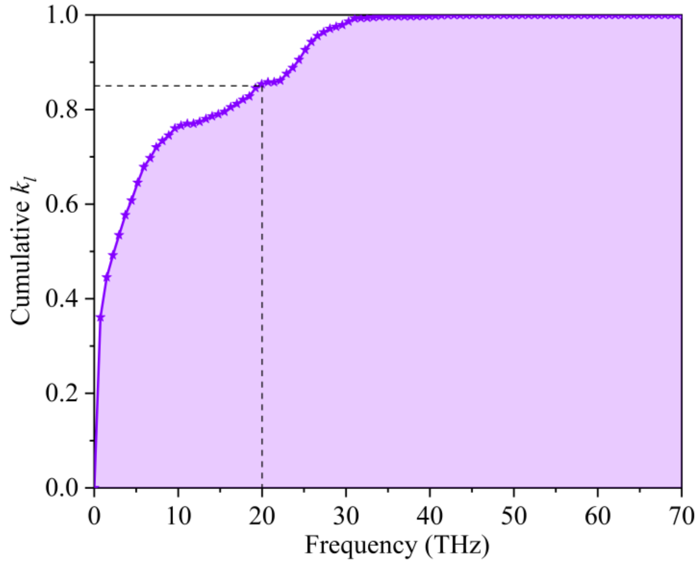

Figure S2. Variation of the normalized cumulative lattice thermal conductivity (k_l) with frequency for ID-GY.
